# Central Dental Association of Northern New Jersey

**Published:** 1893-01

**Authors:** 


### CENTRAL DENTAL ASSOCIATION OF NORTHERN NEW JERSEY.

THE President announced that Dr. Faught's paper was now open for discussion.

(For Dr. Faught's paper, see page 21.)

*Dr. Watkins.*—I anticipate that we shall all profit by this essay. The causes of failure in dental operations are many. One of the most prominent is not being intimately connected with the medical profession. I believe that we would accomplish better results on those we operate if we were nearer the physician, and as a result would be able to do more scientific work.

I believe the profession has not the entire confidence of the

---

public. If we possessed that we would do more scientific work, and the results would be very much better. Consequently, it is the duty of the dentist to educate the public in his neighborhood in regard to the progress made in dentistry. Another way in which we frequently fail is in not having proper material to work with. Take, for instance, teeth that are of a soft, chalky nature, that crumble under the instrument. Ordinarily such teeth are filled with amalgam. Now, in my judgment, amalgam is a very poor material to use. The softer the tooth, the more is amalgam contraindicated. I believe if we were to use the tin or soft gold filling we would do much better. Amalgam will not save a tooth exposed to severe strain, for the amalgam is so much stronger than the tooth that it will outlast it, and the tooth will give way around the filling, whereas if it is a tin or gold filling it will not do so. If the filling is of a soft or somewhat yielding nature the tooth will be apt to last much longer.

Defective manipulation, as you all know, is another cause of failure. It means cutting away all frail walls, making them smooth; the proper disinfecting of the cavity, the proper manipulation of the material, and—not the least by any means—the thorough drying-out of all moisture,—these are the essentials of success. As the essayist has stated, it is not that moisture enters the cavity which is of importance, but the preventing it from remaining there while the filling-material is being inserted. The dentist should also be careful of his physical condition, for if he allows himself to become too tired, and consequently nervous, it is almost impossible for him to do good work. We frequently make mistakes also in not choosing our operations properly,—in not arranging our work and doing that which is the most severe and requires the greatest effort in the early part of the day.

Another reason why we fail, which seems to me of very great importance, is that we are apt to have too many irons in the fire. We should confine ourselves to our legitimate work.

I want to ask Dr. Faught when he closes this discussion to tell us how he would operate with the rubber dam.

*Dr. Ottolengui.*—Mr. President, I do not feel exactly like discussing the paper. I desire to commend the language with which the essayist has clothed his thoughts. I fear this is not attended to as it should be. It too often happens that the editor has to rewrite the papers that are sent to him. Now, it seems to me that those who write should have the elements of writing at their finger-tips.

*Dr. Sanger.*—The paper which we have had read before us to-night is one of very great interest. It is not a paper which you can discuss after a hearty dinner and do it justice. I have one or two things to say. The first is to thank Dr. Faught for his suggestion of putting ourselves into sympathy with our patients; it is a thought that we lose sight of very often. We deal with our patients as if they were so many customers to be furnished with so much merchandise. We forget the patient in the operation that lies before us. The mechanic comes to the front and the scientist takes the background. I think that this is one of the important factors in our failures. It is important for us not only to study the condition of the tooth, the material of which it is formed, and the vitality of the tissue, but also to study the patient himself, or herself, to learn for ourselves his or her temperament. I venture to state that if we will go over in our own minds the operations we have performed during the last week, and ask ourselves how many times we have looked our patient full in the face and considered whether they are nervous or phlegmatic, I think we will find we have neglected something in the operations performed.

Dr. Faught has brought before us a table giving statistics of the temperatures of his patients, and explained his thermometer for taking such temperatures. In this we are not scientific; we are merely mechanics. When we step out from our society we should take our science into every-day practice. If we will do this, it will not be necessary for us to educate the public through the newspapers and periodicals. There is no one better able to measure you than your patient; he will look upon you as either a friend or a foe. He will feel when you are in touch with him, and if you have his confidence you will be able to do more scientific work.

*Dr. Luckey.*—The answer to the subject of the paper, "Causes of Failure," might be a lack of thoroughness. If the operator is thoroughly prepared for his work, the patient is lost sight of in the operation, for the operator is so enthused or so in earnest that he forgets his subject, but this earnestness insures the success of the operation.

*Dr. Bassett.*—One portion of the paper conveys to me a new idea,—the taking of temperatures,—which Dr. Faught has reported, and which will, I think, explain a good deal that we have not before understood. I do not agree with the doctor in his idea of putting ourselves in sympathy with our patients. In fact, if we would forget the individual we might perhaps be capable of doing better work, but if we sympathize with him we are apt to become nervous and in proportion lose our manipulative ability. I think Dr. Faught has covered the entire ground. Dr. Luckey has said that thoroughness is essential to success, and in that statement I quite agree.

*Dr. Roberts.*—I agree with the suggestion advanced by Dr. Faught in the taking of temperatures. When a patient comes into your office nervous and run down with pain, it is almost always necessary, in order to secure the best results, to put yourself in sympathy with him and allay his dread. The true scientific dentist, I take it, is he who can understand and see what is required to be performed.

*Dr. Ottolengui.*—Dr. Luckey did not quite take my idea. I did not mean that unless a man could write well, he must not write, but there is no reason why a man should not write grammatically.

As long as I am up I would say that there is one point that has been raised that has not been discussed, and that is in regard to the treatment of abscesses. It seems to me that it would be well for us to consider this question. The physician is lengthening his hours at his office more and more; the surgeon very rarely goes to the patient's house; all, or I might better say, a great many eminent surgeons, have places in which they can treat their cases. You can have no control of a patient while in his home. I saw an operation last week for removing abscesses, all of which work was done in the theatre of the hospital. The important operations nowadays are not performed at the house of the patient.

*Dr. Boice.*—The paper has not been discussed as to the point made by the essayist of the relation of the dentist to the medical profession. On that point I take issue with Dr. Faught and with his paper.

At the last meeting of dentists in Philadelphia it was suggested that they should have a surgical bureau where the services of a physician could be called for. The measure was referred to the Sanitary Committee, which consisted of five members. Because the physicians said dentistry was not a part of the profession of medicine, the measure was held for over twenty-four hours. The same subject was again brought up, and again defeated because they could not make up their minds whether the bill should read dentists and physicians or physicians and dentists, and it went back to the Sanitary Committee, and they never even read it. Three men agreed with the doctors that the fraternity did not accept the dentists as part of their profession.

In the census report the physicians did not want dentists to be considered as part of the medical profession and registered as such. I think the fact is that dentistry is trying to become a profession. I have had some difficulty over the matter, but without much success. Of course, the dentist has a right to call for the services of a physician, and, if needed, upon a coroner, whichever is the most competent.

*Dr. Faught.*—I want to remark, in the first place, that once, while in the office of a celebrated physician, I saw him taking the temperatures of his patients; the thought then occurred to me why should not this practice be carried out in our profession. Since that time I have been careful to use the same caution, and have put on record in my paper the statistics of temperatures taken in a few cases, and now offer for your inspection the little instrument I use for that purpose.

In reply to Dr. Ottolengui with reference to the treatment of abscess at the house of the physician or in the hospitals, I believe that that is a custom which is coming into general practice not only in this State but in Pennsylvania as well. It also bears out my idea that dentistry cannot be carried on successfully entirely independent of medicine. I am sorry that Dr. Boice has had such an experience with physicians; he certainly has misunderstood the statement made in my paper. I did not say that the dental profession could not do without the services of the medical, but I do think that both professions would do better work were they to fraternize.

If you would commence in the morning by taking the temperatures of patients as they come in, you would be able to do far more satisfactory work. The statistics in the twenty-five cases I have given in my paper were of the temperatures of patients just as I took them coming in for a day or two.

To have a temperature just right is essential to the well-being of the patient. If the temperature falls below the normal, his condition is such as to preclude the possibillty of doing effective work without danger; if a whole degree below normal, it may prove fatal. If you take a patient with a disturbed condition of the nervous system, who is generally "run down," and subject him to a severe operation, you do it at a risk; in fact, it is a wonder that more do not die in the chair. I do not think as many people are injured by the use of nitrous oxide as there are by being operated upon when in a low state of vitality. If dentists took the precaution to examine the condition of their patients, there would be less stigma thrown on the use of anæsthetics.

*Dr. Watkins.*—I asked Dr. Faught a question which he has not answered.

*Dr. Faught.*—I want to say to the doctor that if he will think for a moment he will recollect what I said on this subject. I did not come to put into that paper any particular method, because I use none; and, as I said in my talk, if we would be careful to find out the physical condition of our patients, we would be able to gain better results, and then we can begin to talk about methods of practice.

*The President.*—I wish to thank Dr. Faught personally for his paper, and also to tender him the thanks of the society.

Adjourned.

